# Blockade of IL-17A/IL-17R Pathway Protected Mice from Sepsis-Associated Encephalopathy by Inhibition of Microglia Activation

**DOI:** 10.1155/2019/8461725

**Published:** 2019-10-07

**Authors:** Bo Ye, Tianzhu Tao, Andong Zhao, Liyuan Wen, Xiaofei He, Yi Liu, Qiang Fu, Weidong Mi, Jingsheng Lou

**Affiliations:** ^1^Anesthesia and Operation Center, Chinese PLA General Hospital, Beijing 100853, China; ^2^Department of Anesthesiology, Air Force Medical Center, Beijing 100142, China; ^3^The First Laboratory of Air Force Medical Center, Beijing 100142, China

## Abstract

Sepsis-associated encephalopathy (SAE) is a poorly understood condition that leads to long-term cognitive impairment and increased mortality in survivors. Recent research revealed that IL-17A/IL-17R might serve as a checkpoint in microglia-mediated neuroinflammation. The present study was designed to determine the specific role of IL-17A-mediated microglia activation in the development of SAE. A mouse model of SAE was induced by cecal ligation and puncture (CLP), and behavior performance was evaluated by the inhibitory avoidance test and the open field test. Cytokine expression and microglia activation in brain tissue were determined at 6 h, 12 h, 24 h, 48 h, and day 7 post surgery. Further, septic mice were intracerebral ventricle- (i.c.v.-) injected with recombinant IL-17A, anti-IL-17A ab, anti-IL-17R ab, or isotype controls to evaluate the potential effects of IL-17A/IL-17R blockade in the prevention of SAE. Septic peritonitis induced significant impairment of learning memory and exploratory activity, which was associated with a higher expression of IL-17A, IL-1*β*, and TNF-*α* in the brain homogenate. Fluorescence intensity of Iba-1 and IL-17R in the hippocampus was significantly increased following CLP. Treatment with recombinant IL-17A enhanced the neuroinflammation and microglia activation in CLP mice. On the contrary, neutralizing anti-IL-17A or anti-IL-17R antibodies mitigated the CNS inflammation and microglia activation, thus alleviating the cognitive dysfunction. Furthermore, as compared to the sham control, microglia cultured from CLP mice produced significantly higher levels of cytokines and expressed with higher fluorescence intensity of Iba-1 in response to IL-17A or LPS. Pretreatment with anti-IL-17R ab suppressed the Iba-1 expression and cytokine production in microglia stimulated by IL-17A. In conclusion, blockade of the IL-17A/IL-17R pathway inhibited microglia activation and neuroinflammation, thereby partially reversing sepsis-induced cognitive impairment. The present study suggested that the IL-17A/IL-17R signaling pathway had an important, nonredundant role in the development of SAE.

## 1. Introduction

Sepsis, caused by a dysregulated host response to infection, is the most common cause of Multiple Organ Dysfunction Syndrome (MODS) in the critically ill patients [1]. During sepsis, the central nervous system (CNS) is thought to be one of the first organs affected, which is clinically manifested as sepsis-associated encephalopathy (SAE). As a consequence of systemic inflammatory response to infection, SAE is characterized by diffuse cerebral dysfunction and cognitive impairment but without clinical or laboratory evidence of the direct brain infection, abnormal brain anatomy, encephalorrhagia, or cerebral infarction [2]. The clinical manifestation of SAE can be detected at any stage during sepsis and might appear before the presentation of other systemic features of sepsis. Septic patients with acutely altered mental status were associated with significantly higher mortality rates (49%), as compared to patients with normal mental status (26%) [3, 4].

The pathophysiology of SAE has not been fully established. The proposed mechanisms underlying SAE involved local infiltration of inflammatory cells, brain microvascular endothelial cell dysfunction, disruption of the blood-brain barrier (BBB) and microcirculation, cerebral hypoperfusion, alteration in cerebral neurotransmission, oxidative stress, mitochondrial dysfunction, and apoptosis [5]. Intracerebral inflammation has a crucial role in the pathogenesis of SAE, which is featured by leukocyte infiltration, neuron degeneration, and microglia activation [6]. The permeability of the BBB was increased in septic patients, allowing for the infiltration of peripheral inflammatory mediators in the CNS, which further enhanced the permeability of the BBB and facilitated the production of various inflammatory mediators [6, 7].

Microglia is the most common CNS resident immune cell, and these cells possess the capacity to morphologically and functionally adapt to the ever-changing surrounding microenvironment. Microglial cells are vital participants in CNS development, hemeostasis, and nearly all neuropathological conditions (e.g., stoke, tumors, degenerative diseases, brain injury, and infections) [8]. Microglia rapidly get activated in response to septic challenge, and these cells produced substantial amounts of NO, TNF-*α*, IL-6, IL-1, oxygen-free radicals, and excitatory neurotransmitters. The amplified cerebral inflammatory response exacerbated the neuronal injury, which subsequently impaired the ability of learning and memory and emotional states [9].

IL-17A is the prototype member of the IL-17 family, which is mainly generated by activated CD4+ T cells, CD8+ T cells, NKT cells, *γδ* T cells, and neutrophils. By the interaction with the receptor IL-17R, IL-17A substantially enhanced the inflammatory response and facilitated the recruitment of monocytes and neutrophils to the inflammatory sites [10]. The altered expression of IL-17A and its receptors has been implicated in various CNS inflammatory diseases, such as autoimmune disorder (multiple sclerosis), neurodegenerative diseases (Alzheimer's disease, Parkinson's disease, and epilepsy), hypoxic-ischemia encephalopathy, and posttraumatic brain injury [11]. It has been established that signaling though interaction of IL-17A and IL-17R on microglia could induce the secretion of IL-6, MIP-2, NO, adhesion molecules, and brain-derived neurotrophic factor (BDNF) [12]. Furthermore, activated microglia could produce IL-1*β* and IL-23, which in turn increased the secretion of IL-17A, creating a vicious circle of sustained amplified inflammatory response [13]. In a mouse model of EAE, researchers found that peripheral Th1/Th17 cells were initially recruited to the brain and these cells produced massive IL-17A, mounting the activation of resident microglia and prolonged inflammatory response [14].

Our previous study demonstrated that IL-17A derived from the *γδ* T cells in the peritoneal cavity entered into the circulation rapidly during the early phase of sepsis, and blockade of IL-17A alleviated the proinflammatory response and vital organ injury, thus improving the survival of septic mice. Given the significant role of IL-17A and microglia in the CNS inflammatory diseases, it is important to understand the microglia response to IL-17A/IL-17R signaling in SAE. Hence, the present study was conducted to determine the specific role of IL-17A/IL-17R-mediated microglia activation in the development of SAE. By using the neutralizing antibodies to IL-17A or IL-17R, we hypothesized that blockade of the IL-17A/IL-17R pathway on microglial cells could suppress the potent cerebral inflammatory response, thereby preventing the cognitive impairment during sepsis.

## 2. Materials and Methods

### 2.1. Animals

Experiments were carried out in male C57BL/6 inbred mice (6-8 wk, 20-30 g, Aviation Medical Research Institute, Beijing, China). Mice were maintained on a 12 : 12-hour dark/light cycle, and all efforts were made to ensure the well-being of experimental animals. All procedures were conducted according to the study protocol approved by the Institutional Animal Care and Use Committee of Chinese PLA General Hospital.

### 2.2. Cecal Ligation and Puncture (CLP) Model

A septic mouse model of CLP was made as previously described [15]. Briefly, mice were anesthetized with sevoflurane, and a median laparotomy was made to expose the cecum, which was further ligated at half the distance to the end with a 1-0 Prolene thread. The ligated cecum was punctured twice with an 18G needle, and intestinal content was pushed out. Sham-operated mice underwent the procedure of cecum exposure but without ligation or puncture. The abdominal incision was closed in two layers, and then, mice were resuscitated with 1 ml sterile saline solution by subcutaneous injection. Skin infiltration with 1% lidocaine was performed to minimize the discomfort and pain. Animals had free access to food and water throughout the experiment.

### 2.3. Injection of IL-17A, Anti-IL-17 ab, or Anti-IL-17R ab

To determine the specific role of IL-17A/IL-17R in the development of SAE, mice were randomly divided into 5 groups before CLP and pretreated with the following reagents: recombinant IL-17A (20 ng), anti-IL17A ab (2 *μ*g), anti-IL-17R ab (1 *μ*g), isotype control (isotypeA: IgG1Ƙ 2 *μ*g, isotypeB: IgG2A 1 *μ*g), or saline. All reagents were purchased from eBioscience, San Diego, CA, US, and prepared in 2 *μ*l saline before the intracerebral ventricle (i.c.v.) injection. Mice were anesthetized with sevoflurane, and a stereotactic instrument was used to ensure the high accuracy and reliability.

### 2.4. Cytokine Examination

Cytokines in brain tissue homogenate were determined by ELISA on a microplate reader using the commercial kits at 6 h, 12 h, 24 h, 48 h, and 7 d after CLP. Briefly, mice were deeply anesthetized and euthanized by decapitation. The cerebral hemispheres were rapidly removed, and blood was flushed by saline perfusion. Samples were homogenized with a Potter-Elvehjem homogenizer (Thermo Fisher Scientific Inc.) in Tris-HCl buffer (pH 7.6) containing protease inhibitor cocktail (Nacalai Tesque Inc.). ELISA kits were purchased from R&D, Minneapolis, USA, and all procedures were conducted according to the manufacturer's instructions.

### 2.5. Behavioral Tests

#### 2.5.1. Step Down Inhibitory Avoidance Test

This test was performed to evaluate the learning memory functions as previously described [16]. The apparatus was a 50∗25∗25 cm box containing parallel stainless steel bars spaced 1 cm apart on the floor. A platform (5 cm wide, 2.5 cm high) was placed on the floor of the box. Mice were placed on the platform, and their latency to step down on the grid was measured with an automatic device. During the training session, the animals received a 0.4 mA 2.0 s shock immediately after stepping on the grid. A retention test identical to the training session was performed following CLP but without the electrical stimulation. The step down latency (memory latency) during the test (maximum 180 s) was used to determine the retention of the inhibitory avoidance memory.

#### 2.5.2. Open Field Test

The open field test was carried out to evaluate the motor performance and nonassociated memory. The apparatus was a square plastic box of 60∗60∗50 cm, and the ground of the open field was divided into 9 equal squares by black lines. During the training session, the mice were gently placed on the left rear quadrant and left alone to explore the arena for 5 min. Then, the animals were taken back to their home cage. Mice were submitted again to the same open field following CLP as the test session. Center square entries and line crossing were recorded. A high frequency of these behaviors indicated increased locomotion and exploration and/or a lower level of anxiety.

### 2.6. Immunofluorescence Assay

Immunofluorescence assay was performed using the paraffin sections of the hippocampus. Following deparaffinization and rehydration, the sections were incubated in citrate buffer and then blocked with 3% bovine serum albumin for 30 min. Subsequently, the specimens were incubated with the primary antibodies (anti-Iba1 ab diluted at 1 : 500, anti-CD11b ab and anti-IL-17R diluted at 1 : 100, Abcam, Cambridge, UK) at 4°C overnight and then labeled with the secondary antibodies (CY3-conjugated goat anti-rabbit IgG or FITC-conjugated goat anti-rat IgG, Sigma-Aldrich, St. Louis, MO, USA) diluted at 1 : 400 at 37°C for 30 min. The nucleus was stained with DAPI (Sigma-Aldrich, St. Louis, MO, USA). The sections were dehydrated and mounted for detection by using an inverted fluorescence microscope (Leica, Wetzlar, Germany).

### 2.7. Cell Culture

Primary microglia was prepared and cultured according to the protocols by Lian et al. [17]. Mice were deeply anesthetized and sacrificed by heart puncture. Cortices and hippocampi were collected after removing the meninges from the brain. Homogenous cell suspension was prepared after trituration and then incubation with 2.5% trypsin at 37°C for 15 min. Cell density was counted using a hemocytometer, and cells were seeded at the density of 50,000/cm^2^ for 5-7 days. Astrocytes at the bottom of the flask formed a confluent cell layer, and microglia grew on top of the astrocytic layer. Microglia were collected by vigorously tapping the flasks on the bench top and then cultured at 50,000 cells/cm^2^ in conditioned culture media. The purity of microglia was determined by staining with CD11b and examined by flow cytometry. Microglial cells were pretreated with anti-IL-17R abs or isotype control for 4 hours and then were stimulated with or without the presence of IL-17A (50 ng/ml or 100 ng/ml) or LPS (1 *μ*g/ml). Supernatants were collected at 24 hours, and cytokines (IL-1*β* and TNF-*α*) were measured by using ELISA kits. Cells were collected, and the Iba-1 expression was determined by flow cytometry.

### 2.8. Statistical Analysis

Normal distributed data was presented as the mean ± standard deviation (SD), and nonnormal variables were reported as the median (interquartile range). Difference between groups was determined using the Mann-Whitney test or one-way analysis of variance as appropriate. The Bonferroni-Dunn post hoc test was performed in the analysis of multiple comparisons. All the statistical analyses were conducted by using SPSS software (version 17.0; SPSS Inc., Chicago, IL, US). Graphs were plotted using Prism 6.0 (GraphPad Software, San Diego, CA). A probability value of <0.05 was considered statistically significant.

## 3. Results

### 3.1. Septic Peritonitis Induced Significant Impairment of Learning Memory, Exploratory Activity, and Motility

**Figure 1:**
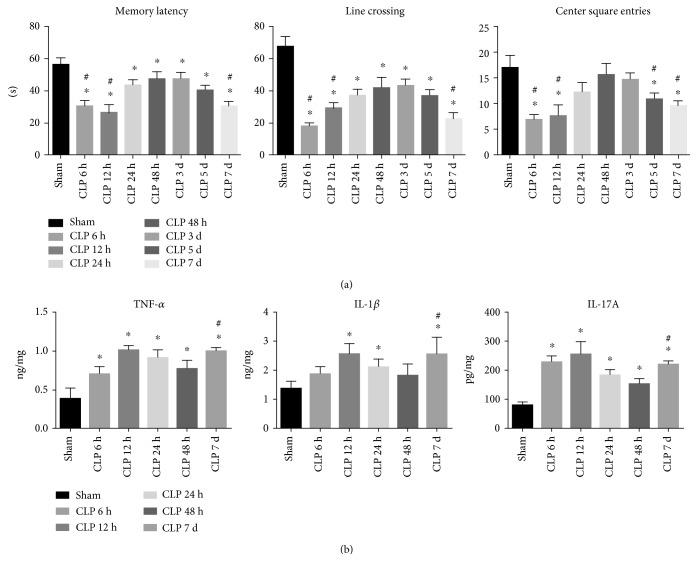
(a) Behavior performance was evaluated by the inhibitory avoidance test and the open field test at various time points following sham or CLP procedures. Each experiment was performed in triplicate and repeated at least twice (*n* = 5‐7 per group). (b) Cytokines of IL-17A, IL-1*β*, and TNF-*α* in brain homogenate were determined by ELISA. ^∗^*P* < 0.05*vs.* sham control, ^#^*P* < 0.05 CLP day 7 *vs.* CLP 48 h, *n* = 5‐6 per group.

The behavioral performance in sepsis survivors was determined at 6 h, 12 h, 24 h, 48 h, day 3, day 5, and day 7 post CLP or sham surgery. In the test section of the inhibitory avoidance task, there was a significant decrease in memory latency time for SAE-induced mice at 6 h and 12 h after procedures. CLP mice had an improved behavioral performance at 24 h, 48 h, and day 3 post surgery, as evidenced by the increased latency time. Interestingly, there was a significantly decreased latency time in CLP mice on day 5 and day 7 as compared to that at 48 h, implying the likelihood of a secondary impairment in the learning memory. In parallel, SAE mice had impaired motor performance and decreased exploratory activity in the early phase, as suggested by low frequency of line crossing and center square entries in the open field test. The locomotion activity and exploratory behavior were largely recovered by day 2, but these animals lost their motility and activity again on day 5 and day 7 (Figure 1(a)).

### 3.2. Sepsis Altered the Expression of IL-17A/IL-17R and Microglia Activation in Brain Tissues

To evaluate the extent of intracranial inflammatory response during sepsis, the expression of IL-17A, IL-1*β*, and TNF-*α* was determined in the brain homogenate following CLP. As shown in Figure 1(b), septic insult substantially increased the expression of IL-17A, IL-1*β*, and TNF-*α* in brain tissues after stimuli. The elevated levels of cytokines peaked at 12 h post surgery and decreased gradually on day 2, but these cytokines significantly increased again on day 7.

**Figure 2:**
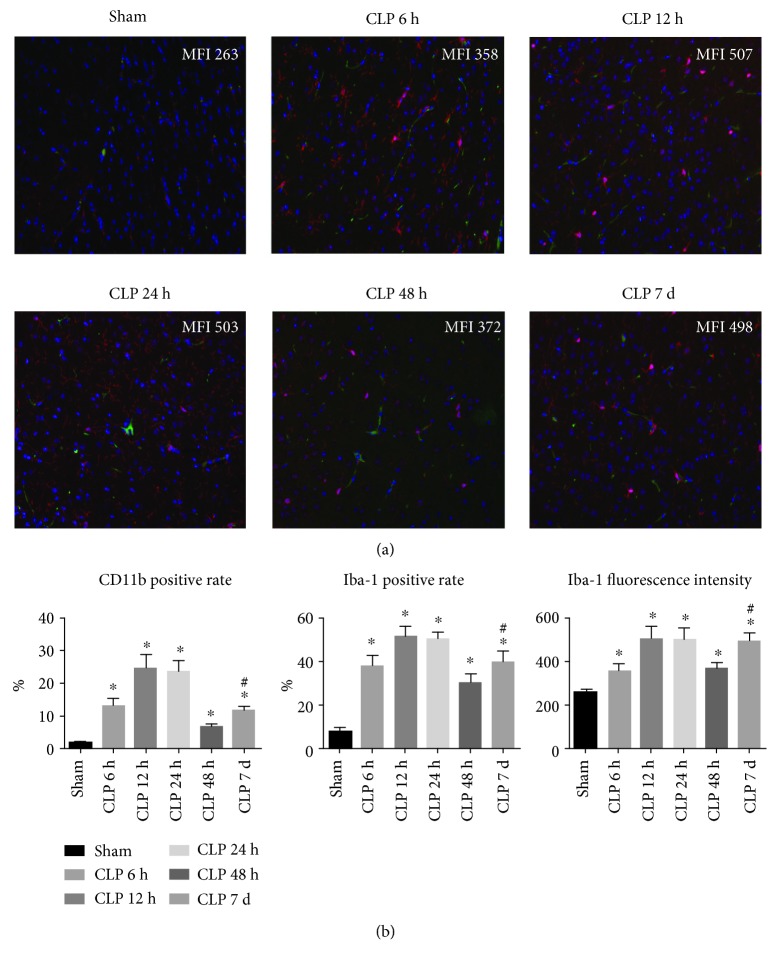
Microglia activation in the hippocampus region was determined by IF at 6 h, 12 h, 24 h, 48 h, and day 7 post surgery. (a) Representative image of the IF assay in the hippocampus. Specimens were stained with CD11b (green), Iba-1 (red), and DAPI (blue). Representative images were merged and analyzed (1 : 200). Mean fluorescence intensity (MFI) of Iba-1 was shown in each image. (b) Summary data of CD11b and Iba-1 expression in the hippocampus region (*n* = 5‐6). ^∗^*P* < 0.05*vs.* sham control; ^#^*P* < 0.05 CLP day 7 *vs.* CLP 48 h.

**Figure 3:**
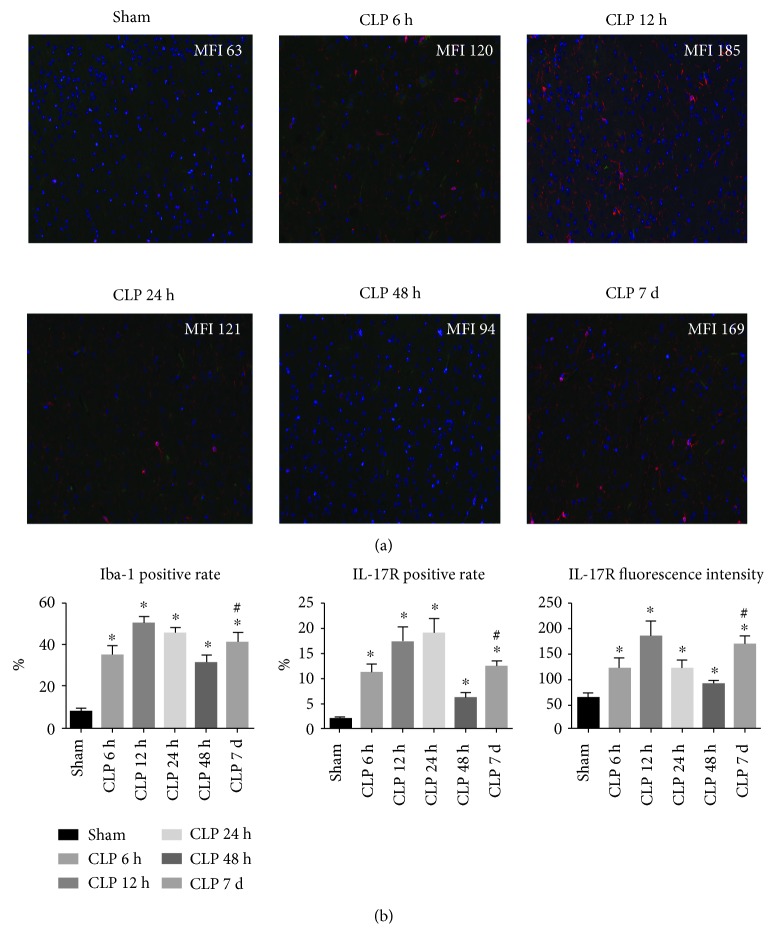
IL-17R expression in the hippocampus region was determined by IF at 6 h, 12 h, 24 h, 48 h, and day 7 post surgery. (a) Representative image of the IF assay in the hippocampus. Specimens were stained with IL-17R (green), Iba-1 (red), and DAPI (blue). Representative images were merged and analyzed (1 : 200). Mean fluorescence intensity (MFI) of IL-17R was shown in each image. (b) Summary data of IL-17R and Iba-1 expression in the hippocampus region (*n* = 5‐6). ^∗^*P* < 0.05*vs.* sham control; ^#^*P* < 0.05 CLP day 7 *vs.* CLP 48 h.

As shown in Figure 2, the percentage of CD11b-positive cells and Iba-1-positive cells as well as Iba-1 fluorescence intensity was increased rapidly following CLP surgery, suggesting that a large amount of microglial cells were activated during the early phase of sepsis. Furthermore, IL-17R expression was determined by the immunofluorescence assay, and we observed an upregulation of IL-17R accompanied by the microglia activation (Figure 3). In accordance with the expression profile of IL-17A in brain homogenate, the expression levels of Iba-1 and IL-17R declined from the peak of 12 h; however, these molecule expression levels rise again on day 7.

### 3.3. Blockade of the IL-17A/IL-17R Pathway Alleviated CNS Inflammatory Response and Cognitive Impairment

**Figure 4:**
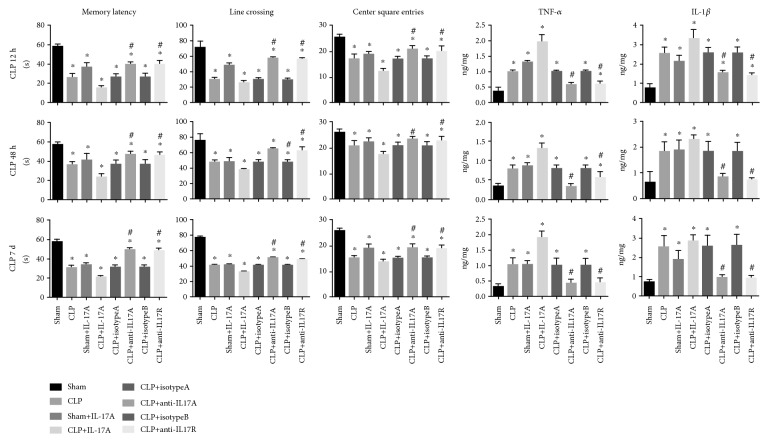
Behavior performance and neuroinflammation were determined following the modulation of the IL-17A/IL-17R pathway. Mice received i.c.v. injection of recombinant IL-17A (20 ng), anti-IL17A ab (2 *μ*g), anti-IL-17R ab (1 *μ*g), isotype control (isotypeA: IgG1Ƙ 2 *μ*g, isotypeB: IgG2A 1 *μ*g), or saline. Samples were collected and analyzed at 12 h, 48 h, or 7 days post surgery. ^∗^*P* < 0.05*vs.* sham control; ^#^*P* < 0.05 (CLP+anti-IL17A ab *vs.* CLP+isotypeA; CLP+anti-IL17R ab *vs.* CLP+isotypeB).

To explore the specific role of the IL-17A/IL-17R pathway in the development of SAE, recombinant IL-17A, anti-IL-17A ab, or anti-IL-17R ab was i.c.v. injected before the CLP surgery. As expected, pretreatment with IL-17A aggravated the behavior performance in SAE-induced mice as evidenced by decreased latency time and lessened frequency of line crossing and center square entries. Importantly, neutralizing anti-IL17A or anti-IL-17R antibodies alleviated the impairment of cognitive function and motility in SAE mice at various time points observed (Figure 4).

**Figure 5:**
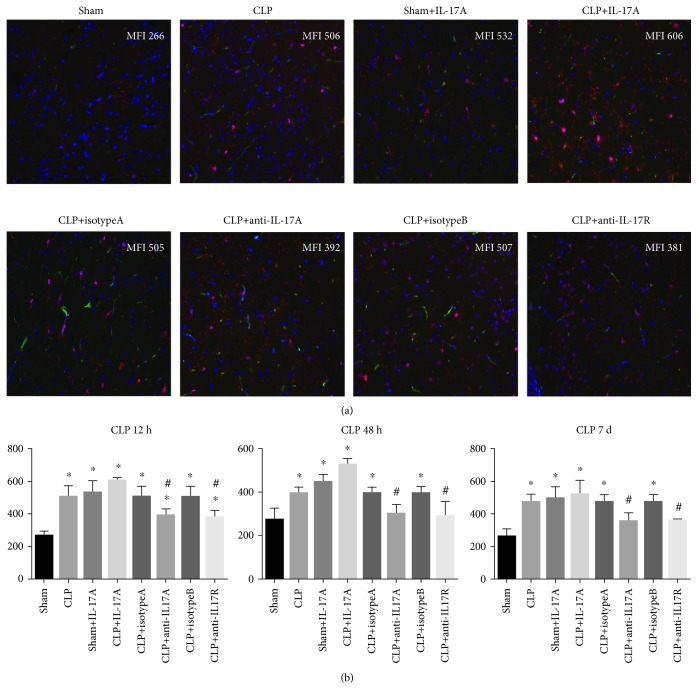
Microglia activation was determined by IF after modulation of the IL-17A/IL-17R pathway. (a) Representative image of the IF assay in the hippocampus at 12 hours post surgery (1 : 200). Specimens were stained with CD11b (green), Iba-1 (red), and DAPI (blue). Mean fluorescence intensity (MFI) of Iba-1 was shown in each image. (b) Summary data of the IF assay at 12 hours, 48 hours, and day 7 post CLP. ^∗^*P* < 0.05*vs.* sham control; ^#^*P* < 0.05 (CLP+anti-IL17A ab *vs.* CLP+isotypeA; CLP+anti-IL17R ab *vs.* CLP+isotypeB).

In accordance with the behavioral performance, pretreatment with recombinant IL-17A enhanced the expression of intracerebral proinflammatory cytokines (IL-1*β* and TNF-*α*) and facilitated the microglia activation in the hippocampus, as indicated by increased Iba-1 fluorescence intensity. In contrast, both anti-IL-17A and anti-IL-17R neutralizing monoclonal antibodies mitigated the CNS inflammation and prohibited the microglia activation (Figure 5).

### 3.4. Activation of Microglia *In Vitro* Was Partially Mediated by the IL-17A/IL-17R Pathway

**Figure 6:**
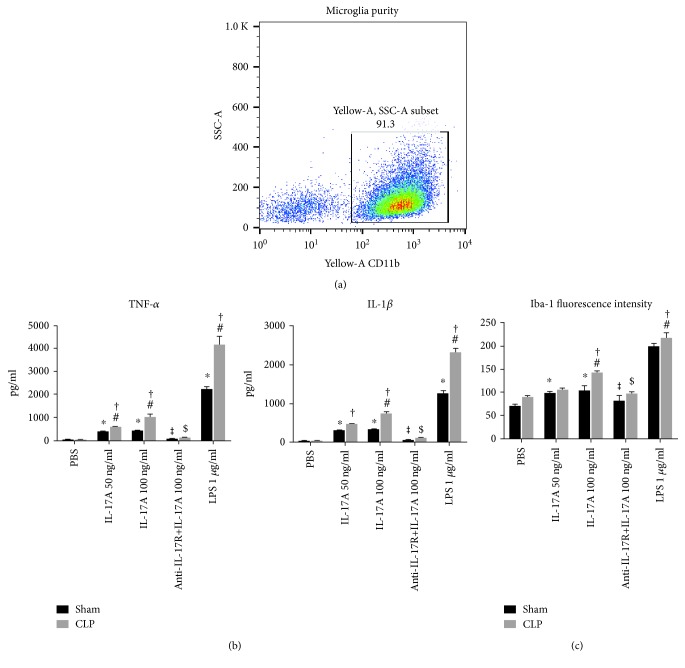
(a) Primary microglial cells were collected and cultured with the purity of over 90%. Cells were stained with anti-CD11b antibody and examined by flow cytometry. (b) IL-17A or LPS induced cytokine production in microglia in vitro. Microglia cultured from CLP or sham-operated mice was pretreated with anti-IL-17R ab or isotype control and then stimulated with or without IL-17A (50 ng/ml or 100 ng/ml) or LPS (1 *μ*g/ml). Supernatant was collected 24 h after stimulation and measured by ELISA. (c) Mean fluorescence intensity of Iba-1 in cultured microglia was determined by flow cytometry. ^∗^*P* < 0.05 vs. PBS control (sham group); ^#^*P* < 0.05 vs. PBS control (CLP group); ^†^*P* < 0.05 CLP vs. sham; ^$^*P* < 0.05 vs. IL-17A 100 ng/ml (CLP group); ^‡^*P* < 0.05 vs. IL-17A 100 ng/ml (sham group).

To test the hypothesis that IL-17A/IL-17R-mediated microglia activation determined the behavioral outcomes in sepsis survivors, primary microglial cells were collected and cultured with or without the presence of recombinant IL-17A. As shown in Figure 6, enrichment of high purity of microglial cells was achieved with the protocol aforementioned. Stimulation with IL-17A or LPS induced significantly higher expression of IL-1*β* and TNF-*α* in cultured microglial cells. As compared to the sham control, microglia harvested from the CLP mice possessed substantially higher capacity to produce inflammatory cytokines under stimuli. Moreover, in line with the enhanced cytokine production, microglia cultured from CLP mice expressed significantly higher levels of Iba-1 under stimulation of IL-17A (100 ng/ml) or LPS (1 *μ*g/ml). Importantly, IL-17A-induced microglia activation and cytokine secretion were largely inhibited by the pretreatment with anti-IL-17R ab, suggesting that the IL-17A/IL-17R signaling pathway had direct effects on the microglia activation.

## 4. Discussion

Sepsis-associated encephalopathy is a common neurological problem but with a serious consequence. Recent studies demonstrated that in patients with bacteremia, 87% had abnormal electroencephalograms (EEG) and 70% were diagnosed with clinical symptoms ranging from lethargy to coma [5, 18]. The survivors frequently displayed prolonged cognitive and behavioral problems [4]. The mechanisms underlying SAE remained undetermined, and the proposed mechanisms involved microvascular disorder, neurotransmitter imbalance, brain microabscess, oxidative stress, apoptosis, and cytokine actions in the brain [2]. In the present study, we found that septic peritonitis induced a significant increase in IL-17A, IL-1*β*, and TNF-*α* in brain homogenate, and the enhanced inflammation was strongly associated with the deterioration in cognitive functions and exploratory activity. Furthermore, blockade of the IL-17A/IL-17R pathway with neutralizing antibodies mitigated the microglia-mediated inflammatory response, thus alleviating the behavioral disorders in septic mice. This is in agreement with the hypothesis that IL-17A/IL-17R-mediated microglia activation is a pivotal step in the development of SAE.

Sepsis induces profound alterations in neuroinflammation, featured by an increase in the adhesion and infiltration of leukocytes in the brain, in the cytokine production, and in the capillary permeability. Disruption of BBB under sepsis is multifactorial, and local cytokines likely play a contributory role in the impairment of BBB integrity [6]. Cytokines (e.g., TNF-*α* and interleukins) can drive the endothelial cell dysfunction by producing massive nitric oxide and oxygen-free radicals [19]. Besides inflammatory response, cytokines in the brain can have direct effects on neuronal excitability. IL-1, released by neutrophils and microglia, can stimulate vagal afferents which in turn affect the brainstem and limbic and hypothalamic structures to produce symptoms such as decreased activity, anorexia, depressed mood, and cognitive impairment [20]. TNF-*α* can alter neurotransmitter metabolism and induce mitochondrial dysfunction. Indeed, administration of TNF-*α* facilitates the metabolism of tryptophan to produce more kynurenine and less serotonin, thereby causing emotional depression [21, 22]. This is in line with the finding in our study that the levels of cytokines in brain homogenate were highly associated with the behavioral performance.

Accumulating evidence is emerging that microglia has an important role in the pathogenesis of SAE. In response to bacterial infections, microglia proliferated and carried out various different functions such as pathogen detection, phagocytosis, and promotion of inflammation [9]. Bacterial components can trigger microglia response through the constitutively expressed pattern recognition receptors such as TLR-2, TLR-4, TLR-9, and nucleotide-binding oligomerization domain-2 (NOD2) [23, 24]. Primed microglia produced exaggerated proinflammatory cytokines (e.g., TNF-*α*, IL-1*β*, and IL-6) and chemoattractants that facilitated the recruitment of circulating leukocytes into the CNS. The increased expression of these mediators in the microenvironment in turn promoted the activation of microglial cells in an autocrine manner, thus amplifying the neuroinflammation [8, 25]. Using minocycline as a pharmacological tool for the inhibition of microglia activation, Michels et al. demonstrated that acute minocycline treatment resulted in a significant decrease in TNF-*α* and IL-6 and prevented the long-term cognitive impairment after sepsis [26]. This is consistent with the present study showing that sepsis stimuli caused substantial activation of microglia in the hippocampus. Moreover, blockade of the IL-17A/IL-17R pathway in vivo largely inhibited the microglia response, which was accompanied by improved memory, exploratory activity, and motor performance. These findings strongly suggested that microglia activation is essential for the development of CNS inflammation and the consequential cognitive dysfunction.

Microglial cells can be activated by a variety of factors, among which IL-17A seems to have an indispensable role in the transformation of microglia phenotype and function [12]. IL-17A, secreted mainly by Th17 cells, is actively involved in mediating proinflammatory response via triggering the expression of IL-1, IL-6, and TNF-*α* in microglia [10]. Our previous study suggested that *γδ* T cells seemed to be the main source of systemic IL-17A during the early stage of sepsis [15]. It is possible that the circulating IL-17A can transfer to the CNS with the presence of BBB impairment. Furthermore, the inflammatory mediators in the brain could activate the innate immune cells in the brain, like Th17 cells and innate *γδ* T cells, which is likely to promote the IL-17A production in brain tissue. With the progression of sepsis, IL-17A can be largely secreted by Th17 cells in the lymphoid tissue. In the present study, we detected a significant increase of IL-17A and the related cytokines (TNF-*α* and IL-1*β*) in brain homogenate following CLP, and the inhibition of the IL-17A/IL-17R pathway largely alleviated the CNS inflammatory response. These findings provided to the support that IL-17A likely served as a major checkpoint in microglia-mediated neuroinflammation.

A recent study revealed that higher levels of IL-17A were associated with poorer cognitive status in subjects with depressive symptoms in ischemic stroke patients [27]. McManus et al. demonstrated that respiratory infection with Bordetella pertussis accelerated cognitive decline in older APP/PS1 mice via promoting the infiltration of IL-17-producing T cells [28]. In the present study, we found that blockade of the IL-17A/IL-17R pathway attenuated the exaggerated cytokine production, thereby reversing the cognitive dysfunction following septic stimuli. Hence, we speculated that IL-17A-driven neuroinflammation seemed to have a nonredundant role in the progression of cognitive dysfunction.

Besides acute symptoms, cognitive dysfunction secondary to sepsis is featured by the long-term cognitive impairments. Abundant data suggested that survivors of sepsis experienced significant deterioration in cognitive and functional status well beyond the acute phase [4, 29]. However, the mechanisms underpinning the long-term consequences remain largely unknown. In the present study, sepsis caused a significant decline in cognitive abilities during 24 hours post surgery. Interestingly, the relevant behavioral performance was substantially improved on day 2, but this performance became progressively worse on day 7. The dynamic change of cognitive function might be interpreted by several aspects. Septic peritonitis induced a rapidly proinflammatory response in the early phase, resulting in the BBB dysfunction, acute neuroinflammation, microglia activation, and neuron metabolism disorders. With the clearance of bacterial products, cytokine production was reduced and the acute CNS dysfunction was largely restored. Meanwhile, septic insult induced a multitude defects in host immunity, and the long-term immunosuppression resulted in the susceptibility to a secondary infection. It is believed that sepsis-induced intestinal barrier dysfunction allowed for the ongoing systemic translocation of bacteria. These microbes might spread to the brain, causing the microabscess, neuroinflammation, and a great loss of neuron. Long-term neurological impairment might also be associated with microcirculatory disturbance, as well as disseminated intravascular coagulation in the brain. The cognitive deficits seemed to be highly associated with the IL-17A/IL-17R-mediated microglia activation, suggesting that IL-17A/IL-17R signaling is at least partially involved in the development of the prolonged cognitive impairment, although further research is still warranted to fully elicit the molecular mechanisms.

Our study has several limitations. First, we determined the expression of IL-17A in brain homogenate and IL-17R expression in the hippocampus, while the expression profile of IL-17A/IL-17R in other specific regions in the brain remained undetermined. Previous research revealed that IL-17A can be produced by Th17, CD4+, CD8+, *γδ* T, invariant NKT, innate lymphoid cells, and neutrophils [30]. Hence, it remained unknown whether the IL-17A in homogenate was mainly produced by the resident brain cells or by the circulating leukocytes. Second, this study was designed to strengthen the role of IL17A/IL17-R in the initiation of microglia activation during SAE; hence, we selected an early time point (before sepsis) to modulate the IL-17/IL-17R pathway. The therapeutic effects of IL-17A/IL-17R blockade at different time points should be further evaluated. Third, we performed the behavioral tests at various time points in a 7-day period, without determining the dynamic alteration of cognitive function in an extended period.

## 5. Conclusions

Sepsis induced high expression of IL-17A/IL-17R and its related cytokines in the brain, which further drove the microglia activation and neuroinflammation. Blockade of the IL-17A/IL-17R pathway with neutralizing antibodies could suppress the CNS inflammation and microglia response, thereby partially reversing sepsis-induced cognitive dysfunction. Our study demonstrated that the IL-17A/IL-17R signaling pathway in microglia had an important, nonredundant role in the development of SAE.

## Data Availability

The data used to support the findings of this study are available from the corresponding authors upon request.
